# Supplementary material for: Testing the practical utility of implicit measures of beliefs for predicting drunk driving
Source: PLoS One. 2022 Sep 29;17(9):e0275328. doi: 10.1371/journal.pone.0275328 (PMC9521934; doi:10.1371/journal.pone.0275328)
Supplement: S1 Appendix — (DOCX) [file pone.0275328.s001.docx]

**S1 Appendix: Exploratory analyses**

**Utility of the A-DUI-IAT to predict past and self-rated future likelihood of drunk driving**

A-DUI-IAT scores were significantly lower for participants without a history of drunk driving (*M* = 0.07, *SD* = 0.36) than for participants who had driven drunk in the past year (*M* = 0.33, *SD* = 0.35), *t*(293.33) = 5.74, *d* = 0.73, *p* < .001, and participants who had driven drunk in the past month (*M* = 0.35, *SD* = 0.35), *t*(190.05) = 5.50, *d* = 0.77, *p* < .001.

The AUC was .70 (95% CI = 0.63-0.76) for past year drunk driving and .71 (95% CI = .64-.78) for past month drunk driving, which is well above chance level (.50). The threshold to maximize sensitivity and retain fair specificity (-0.07 IAT score) produced 88% sensitivity and 39% specificity for detecting past year drunk driving and 89% sensitivity and 39% specificity for detecting past month drunk driving. The threshold to maximize specificity and retain fair sensitivity (0.57 IAT score) produced 87% specificity and 24% sensitivity for detecting past year drunk driving and 87% specificity and 25% sensitivity to detect past month drunk driving.

Higher A-DUI-IAT scores were significantly associated with past year drunk driving, OR = 8.02, 95% CI = [3.73, 18.34], *p* < .001, past month drunk driving, OR = 8.62, 95% CI = [3.72, 21.54], *p* < .001, and self-rated future likelihood of drunk driving, OR = 5.08, 95% CI = [2.38, 11.36], *p* < .001. Results revealed that the A-DUI-IAT showed incremental validity for the prediction of past year drunk driving, χ^2^ = 11.26, *p* <.001 and past month drunk driving, χ^2^ = 8.96, *p* = .003, but not for the prediction of self-rated future likelihood of drunk driving, χ^2^ = 1.92, *p* = .17.

**Sensitivity of P-DUI-IAT to recency and frequency of drunk driving behavior**

We tested whether P-DUI-IAT scores are sensitive to recency and frequency of DUI behaviour by conducting separate ANOVAs with recency (i.e., drunk driving in the past month and drunk driving in the past year, but not in the past month) and frequency of drunk driving as between-subjects factors. Results showed that P-DUI-IAT scores were related to frequency of drunk driving behavior in the past year, *F*(10, 240) = 4.31, *p* < .001, and in the past month, *F*(7, 243) = 4.40, *p* < .001, but not to frequency of drunk driving at follow-up, *F*(8, 206) = 1.86, *p* = .067. Results also showed that there was no effect of recency on P-DUI-IAT scores, *F*(1, 130) = 1.59, *p* = .21.
