# Supplementary material for: Testing the practical utility of implicit measures of beliefs for predicting drunk driving
Source: PLoS One. 2022 Sep 29;17(9):e0275328. doi: 10.1371/journal.pone.0275328 (PMC9521934; doi:10.1371/journal.pone.0275328)
Supplement: S1 Table — (DOCX) [file pone.0275328.s002.docx]

**S1 Table. Category labels and items for the past driving under the influence implicit association test.**

| Category labels | Items |
| --- | --- |
| True | I’m looking at a screen |
|  | I’m doing a computer task |
|  | I’m pressing computer keys |
|  | I’m reading these sentences |
| False | I’m climbing a mountain |
|  | I’m eating in a downtown restaurant |
|  | I’m playing football |
|  | I’m dancing in a club |
| I have drunk driven before | I have driven after I drank alcohol |
|  | Drunk driving is something I have done |
|  | I have driven while being drunk |
|  | I have been drunk when I was driving |
| I have never drunk driven | I have never driven after I drank alcohol |
|  | Drunk driving is something I have not done |
|  | I have always driven while sober |
|  | I have been sober every time I was driving |
