# Supplementary material for: Testing the practical utility of implicit measures of beliefs for predicting drunk driving
Source: PLoS One. 2022 Sep 29;17(9):e0275328. doi: 10.1371/journal.pone.0275328 (PMC9521934; doi:10.1371/journal.pone.0275328)
Supplement: S2 Table — (DOCX) [file pone.0275328.s003.docx]

**S2 Table. Category labels and items for the acceptability driving under the influence implicit association test.**

| Category labels | Items |
| --- | --- |
| True | I’m looking at a screen |
|  | I’m doing a computer task |
|  | I’m pressing computer keys |
|  | I’m reading these sentences |
| False | I’m climbing a mountain |
|  | I’m eating in a downtown restaurant |
|  | I’m playing football |
|  | I’m dancing in a club |
| Drink driving is sometimes acceptable to me | Driving after drinking alcohol is acceptable to me |
|  | If you drive carefully, it is okay to drink and drive |
|  | I’m okay with driving after drinking alcohol |
|  | One can still drive after drinking alcohol |
| Drink driving is never acceptable to me | Driving after drinking alcohol is unacceptable to me |
|  | Under no circumstances, it is okay to drink and drive |
|  | I’m opposed to driving after drinking alcohol |
|  | One should never drive after drinking alcohol |
