# Supplementary material for: Testing the practical utility of implicit measures of beliefs for predicting drunk driving
Source: PLoS One. 2022 Sep 29;17(9):e0275328. doi: 10.1371/journal.pone.0275328 (PMC9521934; doi:10.1371/journal.pone.0275328)
Supplement: S3 Table — (DOCX) [file pone.0275328.s004.docx]

**S3 Table. Risk factors for drunk driving outcomes (Study 1).**

| Variable | DUI since driver’s license OR (95% CI) | Past month DUI OR (95% CI) | Future likelihood DUI OR (95% CI) | Prospective DUI OR (95% CI) |
| --- | --- | --- | --- | --- |
| Gender (male) | 1.77 (0.92, 3.42) | 3.07 (0.94, 11.78) | 1.46 (0.66, 3.22) | 1.62 (0.57, 4.52) |
| Age | 1.06 (0.98, 1.14) | 0.93 (0.68, 1.12) | 1.00 (0.88, 1.09) | 0.95 (0.77, 1.07) |
| Units of alcohol | 1.13*** (1.07, 1.20) | 1.15*** (1.06, 1.24) | 1.09**(1.02, 1.15) | 1.08 (0.97, 1.22) |
| PBC | 2.20*** (1.72, 2.88) | 2.70*** (1.72, 4.59) | 2.14***(1.62, 2.89) | 1.43 (0.95, 2.10) |
| DUI frequency since driver’s license | - | - | 2.50*** (1.85, 3.60) | 1.34* (1.02, 1.88) |
| DUI frequency past month | - | - | 87.47***(14.57, 1691.37) | 8.82 (1.43, 217.83) |

*Note.* PBC = perceived behavioral control; DUI = driving under the influence; OR = odds ratio; CI = confidence interval.

** p* < .05. ** *p* < .01. *** *p* < .001.
