# Supplementary material for: Testing the practical utility of implicit measures of beliefs for predicting drunk driving
Source: PLoS One. 2022 Sep 29;17(9):e0275328. doi: 10.1371/journal.pone.0275328 (PMC9521934; doi:10.1371/journal.pone.0275328)
Supplement: S4 Table — (DOCX) [file pone.0275328.s005.docx]

**S4 Table. Risk factors for drunk driving outcomes (Study 2).**

| Variable | Past year DUI OR  (95% CI) | Past month DUI OR (95% CI) | Future likelihood DUI OR (95% CI) | Prospective DUI OR (95% CI) |
| --- | --- | --- | --- | --- |
| Gender (male) | 2.60*** (1.56, 4.37) | 2.90*** (1.64, 5.22) | 1.86* (1.08, 3.23) | 2.49** (1.36, 4.62) |
| Age | 0.97** (0.95, 0.99) | 0.97* (0.95, 0.99) | 0.98* (0.95, 0.10) | 0.99 (0.97, 1.01) |
| Units of alcohol | 1.07*** (1.03, 1.10) | 1.06*** (1.03, 1.10) | 1.04*** (1.02, 1.07) | 1.05*** (1.02, 1.08) |
| PBC | 2.04*** (1.69, 2.52) | 2.28*** (1.85, 2.89) | 2.41*** (1.95, 3.041) | 1.92*** (1.59, 2.37) |
| DUI frequency past year | - | - | 1.79*** (1.54, 2.13) | 2.11*** (1.73, 2.70) |
| DUI frequency past month | - | - | 5.40*** (3.36, 9.26) | 6.76*** (3.76, 13.31) |

*Note.* DUI = Driving under the influence; OR = odds ratio; CI = confidence interval.

* *p* < .05. ** *p* < .01. *** *p* < .001.
